# Where is the friend's home?

**DOI:** 10.3389/fgene.2014.00400

**Published:** 2014-11-13

**Authors:** Guo-Bo Chen

**Affiliations:** Centre of Neurogenetics and Statistical Genomics, Queensland Brain Institute, The University of QueenslandSt. Lucia, QLD, Australia

**Keywords:** friendship, nature selection, Framingham heart study, FST statistics, GWAS

Using Framingham Heart Study (FHS) data (accession no. phs000153.v7.p6), Christakis and Fowler (2014) showed that friends had more extended genetic correlation than strangers had. This finding may suggest that friends are “functional kin.” Depending on the context, functional kin may have high genetic similarity (homophilic) if people have similar functional kin as friends; conversely, functional kin may have low/negative genetic similarity (heterophilic) if people have complementary functional kin as friends. Christakis and Fowler reported that between friends there is positive/negative genetic correlation at the single nucleotide polymorphism (SNP) level, indicating heritability of friendship. This result is very interesting, particularly for the social sciences (Skyrms et al., 2014), but deserves scrutiny. This note demonstrates that (1) high genetic similarity between friends is possible if a person finds friends from his/her own cultural background, which is a surrogate for genetic similarity, or (2) low genetic similarity is possible if a person finds friends from a different cultural background. As illustrated below, the mechanism involved in inflating/deflating genetic similarity between friends is analogous to the way population stratification raises the false positive rate in case-control studies.

In Christakis and Fowler's study, a single-marker regression was conducted to test the association for friendship. The regression is defined as

$$g_{e.m}=\mu_{m}+b_{m}g_{f.m}+e_{m}$$

in which *g_e.m_* and *g_f.m_* are the genotypes of “ego” and his/her friend, respectively, at the *m^th^* locus (*m* = 1, 2, 3 … *M*). The regression coefficient is estimated as ${\hat{b}}_{m}=\frac{cov(g_{e.m},g_{f.m})}{var(g_{f.m})}$. In a conventional GWAS regression, the phenotype is the same for each locus, whereas in this regression, the *g_e.m_* phenotype is updated to match each *g_f.m_* locus. However, this actually models *F_st_* under the circumstances as discussed below (blue box in Figure 1). For clarity, the subscript *m* is hereafter omitted.

**Figure 1:**
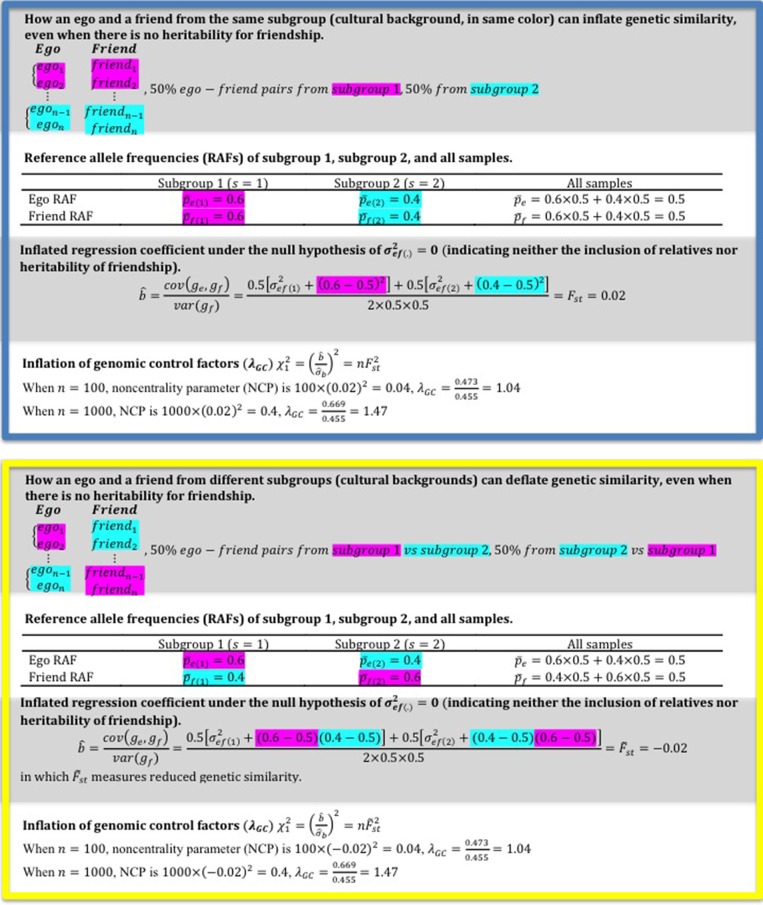
**Illustration of how an ego-friend from the same/different subgroup(s) may inflate/deflate genetic similarity as indicated by λ_*GC*_**.

Assume that in the sample there are *S* subgroups (e.g., *S* = 2), each of which has *n_s_* individuals. The proportion of the *s^th^* subgroup to the total sample size is $w_{s}=\frac{n_{s}}{n}$, in which $n=\sum_{s\;=\;1}^{S}n_{s}$. Of the total *n* ego-friend pairs, *n_s_* are from the *s^th^* subgroup. The variance of *g_f_* can be written as the within-group variance and the between-group variance (http://en.wikipedia.org/wiki/Mixture_distribution)

$$var\left(g_{f}\right)=\sum_{s=1}^{S}w_{s}[\sigma_{f(s)}^{2}+{\left({\bar{p}}_{f(s)}-{\bar{p}}_{f}\right)}^{2}]$$

in which *p*_*f*(*s*)_ is the reference allele frequency (RAF) of the friends from the *s^th^* subgroup. *p_f_* = ∑ *w_s_p*_*f*(*s*)_ is the RAF of the friends, σ^2^_*f*(*s*)_ = 2*p*_*f*(*s*)_(1 − *p*_*f*(*s*)_) is the within-sub-population variance of the friends, and (*p*_*f*(*s*)_ − *p_f_*)^2^ is the between-sub-population sampling variance. Similarly, the covariance between *g_e_* and *g_f_* can be written as

$$\begin{array}{l} cov\left(g_{e},g_{f}\right)=\sum_{s=1}^{S}w_{s}[\sigma_{ef\left(s\right)}^{2}\\ \;+\;({\bar{p}}_{e(s)}-{\bar{p}}_{e})({\bar{p}}_{f(s)}-{\bar{p}}_{f})] \end{array}$$

in which σ^2^_*ef*(*s*)_ is the covariance between the friends' genotypes within the *s*^*th*^ subpopulation, and (*p*_*e*(*s*)_ − *p_e_*)(*p*_*f*(*s*)_ − *p_f_*) is the between-sub-population covariance. σ^2^_*ef*(*s*)_ ≠ 0 if the pair of friends is related or there is heritability for friendship at the *i^th^* locus.

In Christakis and Fowler's study, the null hypothesis is that *H*_0_ : σ^2^_*ef*(*s*)_ = 0 (i.e., no heritability for friendship). Even if friendship is not heritable and relatives are not included, friends can still have inflated/deflated genetic correlation, which raises the regression coefficient from zero, if an ego finds friends from the same/different cultural background.

If an ego finds friends from the same cultural background, *p*_*e*(*s*)_ will be similar to *p*_*f*(*s*)_. Assuming that *p*_*e*(*s*)_ = *p*_*f*(*s*)_, the regression coefficient can be written as

$$\begin{array}{l} \hat{b}=\frac{cov(g_{e},g_{f})}{var(g_{f})}\\ =\frac{\sum_{s=1}^{S}w_{s}({\bar{p}}_{e(s)}-{\bar{p}}_{e})({\bar{p}}_{f(s)}-{\bar{p}}_{f})}{\sum_{s=1}^{S}w_{s}[\sigma_{f(s)}^{2}+{\left({\bar{p}}_{f(s)}-{\bar{p}}_{f}\right)}^{2}]}\\ \approx \frac{\sum_{s=1}^{S}w_{s}{\left({\bar{p}}_{f\left(s\right)}-{\bar{p}}_{f}\right)}^{2}}{\sum_{s=1}^{S}w_{s}[\sigma_{f\left(s\right)}^{2}+{\left({\bar{p}}_{f\left(s\right)}-{\bar{p}}_{f}\right)}^{2}]} \end{array}$$

By definition, $F_{st}=\frac{\Sigma_{s=1}^{S}}{w_{s}}{\left({\bar{p}}_{f(s)}-{\bar{p}}_{f}\right)}^{2}var\left(g_{f}\right)\geq \text{0}$. The standard deviation of $\hat{b}$ is ${\hat{\sigma }}_{b}=\sqrt{\frac{\sigma_{g_{e}}^{2}}{n\sigma_{g_{f}}^{2}}}\approx \sqrt{\frac{1}{n}}$.

Given $\hat{b}$ and $\hat{\sigma }$_*b*_, a *z*-score test can be constructed as $z=\frac{\hat{b}}{{\hat{\sigma }}_{b}}=\sqrt{n}F_{st}$ and *z*^2^ ~ χ^2^_1_, with the non-centrality parameter (NCP) Δ = *nF*^2^_*st*_. Christakis and Fowler reported λ_*GC*_, the genomic inflation factor (Devlin and Roeder, 1999), for their GWAS analysis. In their study, $\lambda_{GC}=\frac{median(\chi_{1}^{2})}{0.455}=1.04$, which meant that the median of the observed χ^2^_1_ value was 0.473, with NCP Δ = *nF*^2^_*st*_ = 0.04. Thus, the median of *F_st_* was ${\hat{F}}_{st}=\sqrt{0.04/n}=0.0066$ between the egos and the friends (*n* = 907 in Christakis and Fowler's study).

An early study (Cavalli-Sforza et al., 1996) indicated that *F_st_* = 0.016 for European descendants, and a recent study (Novembre et al., 2008) using high-density SNPs showed that *F_st_* = 0.004 between European nations (the estimated mean of *F_st_* = 0.0042 over nearly 900 thousand common HapMap SNPs between CEU and TSI). Although the estimate of *F_st_* depends on other factors, such as ascertainment and the sample proportion for each subgroup (Bhatia et al., 2013), the estimated $\hat{F}$_*st*_ between the egos and the friends seems to fall in-between the aforementioned values. For a loosely defined trait such as friendship, the heritability may be low, if not zero. However, as long as friendship is more frequently established within one's cultural background, *F_st_* will inflate genetic similarity among friendships even in the absence of heritability/“functional kin.” With an increased sample size, λ_*GC*_ *will be even larger* (blue box in Figure 1), a phenomenon that resembles the manner in which population stratification inflates type I error rate in GWAS.

In the discussed study, the negative genetic correlation between friends was also highlighted. Similarly, if an ego finds friends from a different cultural background, the regression coefficient will tend to turn negative (denoted as $\tilde{F}$_*st*_, indicating reduced genetic similarity between friends, see the yellow box in Figure 1). In practice, both patterns, not as extreme as demonstrated though, will be possible between friends and lead to homophily and heterophily as observed.

In their GWAS-like analysis, principle components were used as covariates to control for *F_st_*. However, covariates may not completely eliminate the background effects, such as *F_st_*. When covariates reduce *F_st_*, the heritability of friendship will also be reduced, potentially to zero. Although the analysis in this note does not entail the rejection of the conclusion drawn by Christakis and Fowler, it warns against misleading interpretations of the results.

## Conflict of interest statement

The author declares that the research was conducted in the absence of any commercial or financial relationships that could be construed as a potential conflict of interest.
